# Using machine learning to predict estimated glomerular filtration rate in type 2 diabetes patients: A 4 year-follow-up study

**DOI:** 10.1097/MD.0000000000044732

**Published:** 2025-10-03

**Authors:** Fang-Yu Chen, Dee Pei, Chun-Heng Kuo, Li-Ying Huang, Mao-Jhen Jhou, Yao-Jen Liang

**Affiliations:** a Division of Endocrinology and Metabolism, Department of Internal Medicine, Fu Jen Catholic University Hospital, School of Medicine, College of Medicine, Fu Jen Catholic University, New Taipei, Taiwan, ROC; b Graduate Institute of Applied Science and Engineering, Fu Jen Catholic University, New Taipei City, Taiwan, ROC; c Department of Medical Education, Fu Jen Catholic University Hospital, School of Medicine, College of Medicine, Fu Jen Catholic University, New Taipei, Taiwan, ROC; d Graduate Institute of Business Administration, Fu Jen Catholic University, New Taipei City, Taiwan, ROC; e Department and Institute of Life-Science, Fu Jen Catholic University, New Taipei, Taiwan, ROC.

**Keywords:** estimated glomerular filtration rate, machine, learning, type 2 diabetes

## Abstract

The global prevalence of type 2 diabetes mellitus (T2D) has been increasing dramatically as well as diabetic kidney disease (DKD). We aimed to compare the accuracies of 4 machine learning (Mach-L) methods with multiple linear regression (MLR) in predicting future estimated glomerular filtration rate (eGFR) in T2D patients and to rank the importance of DKD risk factors. The study was conducted from 2013 to 2019. Nine hundred and seven T2D patients were followed up for 4 years. Data of potential DKD risk factors were collected and calculated. We used 4 different Mach-L methods to predict the eGFR, including classification and regression tree, random forest, artificial neural network, and eXtreme Gradient Boosting. Simple correlation was applied to overview the relationships between baseline risk factors and eGFR at the end of follow-up (eGFR_end_). Besides, traditional MLR was used as a benchmark to evaluate if Mach-L methods could outperform MLR. For model interpretability, Shapley additive explanation was applied to explain the contribution of each feature and directions of impacts in the prediction model. In 4 different Mach-L methods, random forest, classification and regression tree, and eXtreme Gradient Boosting were more superior than MLR in the prediction of the eGFR_end_. The first 6 important risk factors in predicting diabetic eGFR_end_ were body mass index (BMI), baseline high-density lipoprotein cholesterol (HDL-C), baseline urine microalbumin creatinine ratio (MCR), baseline low-density lipoprotein cholesterol (LDL-C), duration of diabetes, and age. By applying Shapley additive explanation, it appeared that age, duration of diabetes, HDL, and LDL were positively related to eGFR_end_ and BMI and MCR were negatively related to eGFR_end_. Mach-L methods were proved to be more accurate in predicting eGFR_end_ than traditional MLR. BMI presented the most influential factor for eGFR_end_, followed by HDL-C, baseline urine MCR, LDL-C, duration of diabetes, and age. These findings highlight the potential of Mach-L to enhance early risk stratification for DKD, enabling timely interventions to preserve renal function in T2D patients.

## 1. Introduction

The global prevalence of type 2 diabetes mellitus (T2D) has been increasing dramatically. It is estimated that 425 million people will have T2D between 2017 and 2045, with most residing in low- and middle-income countries worldwide.^[1]^ In Taiwan, the prevalence increased from 1.3 to 2.2 million between 2005 and 2014, reflecting a pattern consistent with global trends. According to the National Health Insurance Research Database, the prevalence increased from 1.3 to 2.2 million from 2005 to 2014.^[2]^ In 2024, T2D was the top 6th cause of death according to the Taiwan Ministry of Health and Welfare. There were 12,289 T2D died in 2022, which was 5.8% of the total deaths.^[3]^ Parallel with the prevalence, the incidence of diabetes mellitus complication also increased, particularly diabetic kidney disease (DKD).^[4]^ According to The United States Renal Data System, Taiwan has the highest percentage of treated end-stage renal disease, that is, 3587 cases per million populations are under dialysis.^[5]^ This causes a huge burden to both the patients, their families, and also the health providers. The annualized costs for care of dialysis patients in Taiwan averaged US $25,576 per patient-year. This is about twice the price in most of the western countries.^[6]^ Therefore, early detection of the DKD and begin appropriate intervention are of importance to correct this derangement.

Given the importance of DKD, there have been vast amount of studies tried to solve this issue and there were many risk factors identified in T2D. In a recent review, Tziomalos et al divided these factors into modifiable and non-modifiable risk factors. Increased urinary albumin excretion, poor glucose control, high blood pressure, dyslipidemia, overweight, and smoking are modifiable and duration of diabetes, age, and female sex are non-modifiable risk factors.^[7]^ In the same time, some other new risk factors were also proposed by different research groups such as oxidative stress, chronic inflammation (modifiable), genetic background, ethnicity, and glomerular hyperfiltration (non-modifiable).^[7]^

However, it is interesting to note that most of these studies used the traditional statistics such as multiple linear regression (MLR) or logistic regression to analyze the data.^[8,9]^ Recently, the newly developed machine learning (Mach-L) methods have been used widely in the field of medical researches. Mach-L methods apply computer algorithms that can improve automatically through experience and by the use of data.^[10]^ It enables machines to learn from the past data or experiences without being explicitly programmed. It is now become a new modality for data analysis competitive to traditional MLR.^[11–13]^ Mach-L could capture nonlinear relationships and complex interactions among multiple predictors. Moreover, it does not need hypothesis such as linearity and normal distribution. Thus, it has the potential to outperform conventional MLR in diseases prediction.^[13]^

The main goal of the present study is not only to compare the accuracies of 4 Mach-L methods with MLR in predicting future estimated glomerular filtration rate (eGFR) in T2D patients, but also to rank the importance of the risk factors in a Chinese T2D cohort followed up for 4 years and further determining the positive and negative impacts of each feature.

## 2. Method

**Figure 1.**
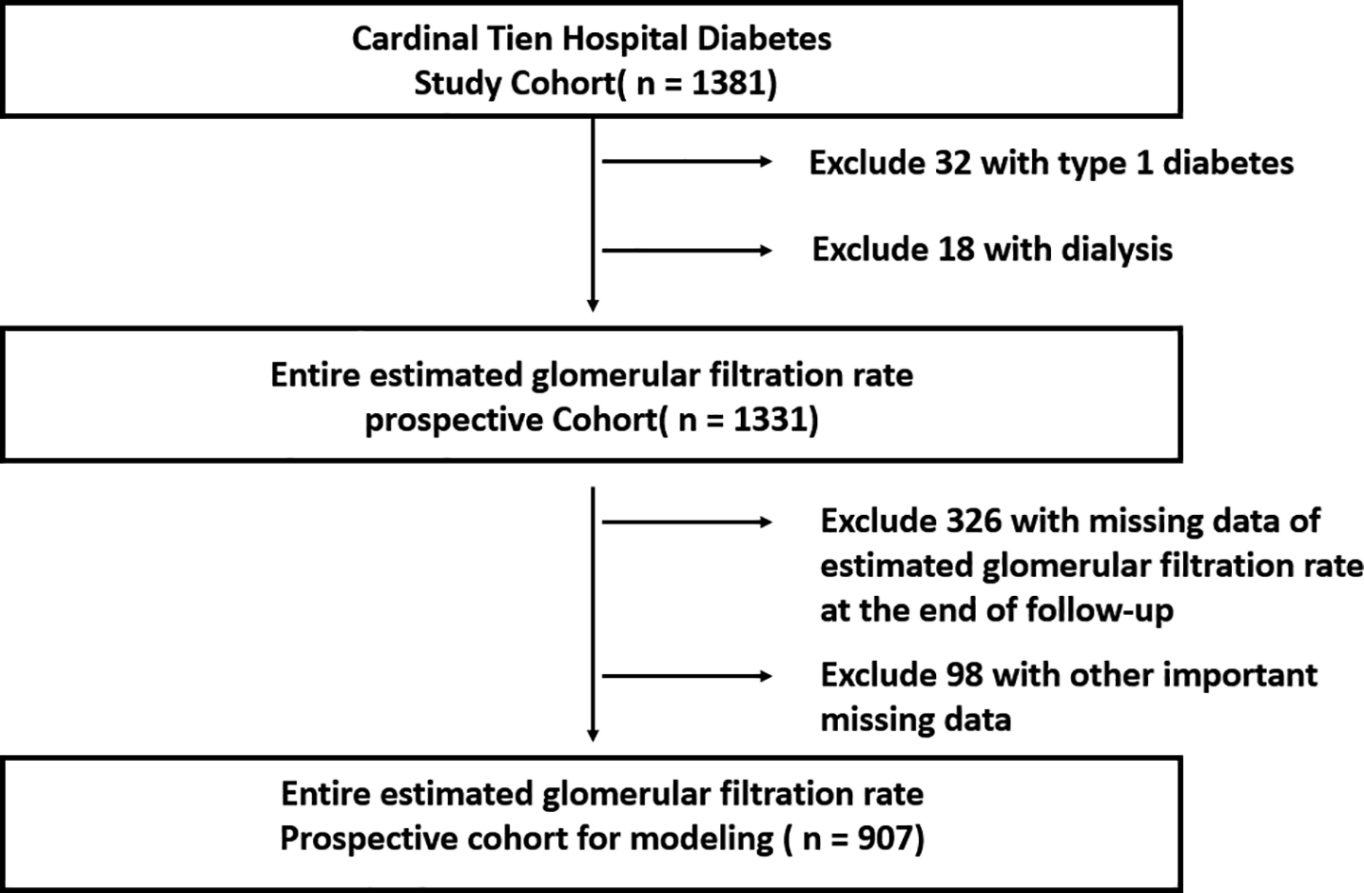
Flow diagram of sample selection. The figure shows the inclusion and exclusion process used to identify the final study cohort.

This study was conducted within the diabetic outpatient clinic in Cardinal Tien hospital in Taiwan from 2013 to 2019. Each subject was given informed consent with the data collected anonymously. The study protocol was approved by the institutional review board of the hospital (IRB no: FJUH113442). Initially, there were 1381 T2D patients enrolled and complete data were collected in 907 T2D (men: 480, women: 427) patients. They were followed up for 4 years. The following inclusion criteria were applied to this study cohort: T2D; aged between 50 and 75 years old; body mass index (BMI) between 22 and 30 kg/m^2^; glycated hemoglobin (HbA1c) between 6.5% and 10.5%; they were not under regular dialysis. The flowchart of population selection is shown in Figure 1.

On the day of the study, a senior nursing staff member recorded the subject’s medical history, including information on any current medications, duration of diabetes, alcohol history, smoking history, and a physical examination was performed. Both systolic blood pressure and diastolic blood pressure were measured by standard mercury sphygmomanometers on the right arm of each subject while seated. BMI was calculated as the subject’s body weight (kg) divided by the square of the subject’s height (m). At the end of following up, the same procedures were done again.

After fasting for 10 hours, blood samples were drawn for biochemical analysis. Plasma was separated from the blood within 1 hour of collection and stored at −30°C until analysis for fasting plasma glucose and lipid profiles. Fasting plasma glucose was measured using a glucose oxidase method (YSI 203 glucose analyzer, Yellow Springs Instruments, Yellow Springs). Total cholesterol and triglyceride levels were measured using a dry, multilayer analytical slide method with the Fuji Dri-Chem 3000 analyzer (Fuji Photo Film, Tokyo, Japan). Serum high-density lipoprotein cholesterol (HDL-C) and low-density lipoprotein cholesterol (LDL-C) concentrations were analyzed using an enzymatic cholesterol assay, following dextran sulfate precipitation. Creatinine measurement used the Beckman Coulter AU 5800 biochemical analyzer to determine the blood creatinine by kinetic modified Jaffe method. The eGFR was calculated by Cockcroft–Gault equation ([(140 − Age) × body weight]/[(72 × sCr)] × (0.85 if female)).

Part of the following context in methods were published by our group previously.^[14]^ We use 4 different Mach-L methods to predict the eGFR, namely classification and regression tree (CART), random forest (RF), artificial neural network (ANN), and eXtreme Gradient Boosting (XGBoost). Simple correlation was applied to overview the relationships between baseline risk factors and eGFR at the end of follow-up (eGFR_end_). Besides, traditional MLR was used as a benchmark to evaluate if Mach-L methods could outperform MLR.

As the first method, CART is a tree structures method.^[15]^ It is composed of root nodes, branches, and leaf nodes that based on the tree structures grow recursive from the root nodes and split at each node based on the Gini index to produce branches and leaf nodes with the rule. Then pruning node in the overgrown tree for optimal tree size by using cost-complexity criterion, final to generate different decision rules to compose a complete structures tree.^[16,17]^

RF is the second method of this study, is an ensemble learning decision trees algorithm of combining bootstrap resampling and bagging.^[18]^ RF’s principle is randomly generating many different and unpruned CART decision trees which the decrease Gini impurity is regarded as the splitting criterion, and by all generating trees combined to a forest. Then all of the trees of the forest were averaged or voted to generate output probabilities and a final model that gives to generate a robust model.^[19]^

For the third method, ANNs are biologically inspired computer programs designed to simulate the way in which the human brain processes information. It is a computational model formed from hundreds of single unit artificial neurons which consists of multiple layers of simple processing elements, connected with coefficients (weights) which constitute the neural structure. The neuron performs 2 functions, namely, collection of inputs & generation of an output and then gather their knowledge by detecting the patterns and relationships in data and learn through experience, not from programming.^[20]^

XGBoost is the last Mach-L of this study. It is a gradient boosting technology based on stochastic gradient boosting-optimized extension.^[21]^ Its principle is training many weak models sequentially to ensemble them using the gradient boosting method of outputs which achieve better prediction performance. In the XGBoost, Taylor binomial expansion was used to approximate the objective function and arbitrary differentiable loss functions to make accelerate the model construction converge process.^[22]^ Then, XGBoost applies regularized boosting technique to penalize the complexity of the model and correct the overfitting and thus increase the model accuracy.^[21]^

**Figure 2.**
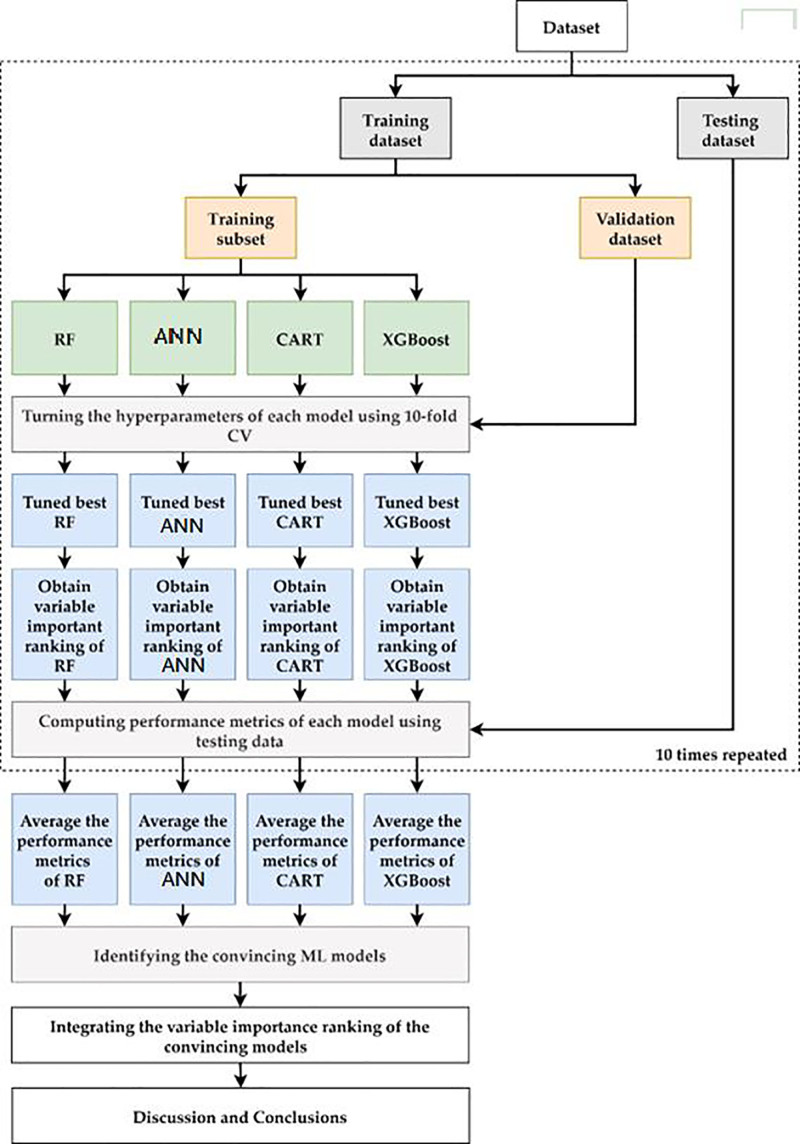
Proposed machine learning model prediction scheme. The diagram illustrates the workflow of machine learning algorithms applied in the analysis, including classification and regression tree (CART), random forest (RF), artificial neural networks (ANN), and eXtreme Gradient Boosting (XGBoost).

The flowchart of the proposed scheme combining the 4 Mach-L methods is demonstrated in Figure 2. As Figure 2 shows, in the proposed scheme, we first collected patients to prepare the dataset for model construction, then the dataset was randomly split into 80% training dataset for model building and 20% testing dataset for out of sample testing. In the training process, each Mach-L method has its own hyperparameters to be tuned for constructing a relatively well performed model. We used a 10-fold cross-validation technique for hyperparameter tuning. To do this, the training dataset was further randomly divided into the training dataset to build the model with a different set of hyperparameters, and the validation dataset for model validation. All possible combinations of hyperparameters were investigated by grid search. The model with the lowest root mean square error on the validation dataset was viewed as the best model of each ML method. The turned best model RF, ANN, CART, and XGBoost are generated and the corresponding variable importance ranking information can be obtained.

In the testing process, the testing data set is used to evaluate the predictive performance of the best RF, ANN, CART, and XGBoost models. We used 5 performance metrics, including mean absolute percentage error, symmetric mean absolute percentage error, relative absolute error, root relative squared error, and root mean squared error to evaluate each method’s prediction error. The performance metrics are shown in Table 1.

**Table 1:** Equations of performance metrics used to evaluate prediction models.

Metrics	Description	Calculation
MAPE	Mean absolute percentage error	$MAPE=\frac{1}{n}\sum_{i=1}^{n}\frac{y_{i}-{\hat{y}}_{i}}{y_{i}}\times 100$
SMAPE	Symmetric mean absolute percentage error	$SMAPE=\frac{1}{n}\sum_{i=1}^{n}\frac{y_{i}-{\hat{y}}_{i}}{(y_{i}+{\hat{y}}_{i})/2}\times 100$
RAE	Relative absolute error	$RAE=\sqrt{\frac{\sum_{i=1}^{n}{\left(y_{i}-{\hat{y}}_{i}\right)}^{2}}{\sum_{i=1}^{n}{\left(y_{i}\right)}^{2}}}$
RRSE	Root relative squared error	$RRSE=\sqrt{\frac{\sum_{i=1}^{n}{\left(y_{i}-{\hat{y}}_{i}\right)}^{2}}{\sum_{i=1}^{n}{\left(y_{i}-\bar{y}\right)}^{2}}}$
RMSE	Root mean squared error	$RMSE=\sqrt{\frac{1}{n}\sum_{i=1}^{N}{\left(y_{i}-{\hat{y}}_{i}\right)}^{2}}$

The table summarizes the formulas for mean absolute percentage error (MAPE), symmetric mean absolute percentage error (SMAPE), relative absolute error (RAE), root relative squared error (RRSE), and root mean squared error (RMSE). ${\hat{y}}_{i}$ and $y_{i}$ represent predicted and actual values, respectively; n stands the number of instances.

In order to provide a more robust comparison, the training and testing process mentioned above is randomly repeated 10 times. The averaged metrics of RF, ANN, CART, and XGBoost models were used to compare the model performance of the benchmark; MLR model used the same training and testing dataset with the Mach-L methods. A Mach-L model with an average metric lower than that of MLR is considered as the convincing model. In the present study, CART, RF, and XGBoost outperformed linear regression. With different modeling characteristics, different Mach-L methods may produce different variable importance percentages. We integrated the variable predictor importance percentages of the 3 Mach-L and re-rank them for the overall importance. In the final stage of the proposed scheme, we summarized and discussed our significant findings about convincing Mach-L models and identified important variables.

In this study, all methods were performed with R software version 4.0.5 and RStudio version 1.1.453 with the required packages installed (http://www.R-project.org; https://www.rstudio.com/products/rstudio/). The implementations of RF, ANN, CART, and XGBoost are, respectively, “randomForest” R package version 4.6-14,^[23]^ “gbm” R package version 2.1.8,^[24]^ “rpart” R package version 4.1-15,^[25]^ and “XGBoost” R package version 1.5.0.2.^[26]^ As well as, to estimate the best hyperparameters set for developed effective CART, RF, ANN, XGBoost methods, the “caret” R package version 6.0-90 was used.^[27]^ The logestic regression was implemented by the “stats” R package version 4.0.5; the default setting was used to construct the models.

SPSS version 19.0 (IBM Inc., Armonk) was used to perform the traditional MLR. On the other hand, CART, RF, ANN, and XGBoost were calculated by R. Data are presented as mean ± standard deviation (SD). All data were tested for normal distribution with Kolmogorov–Smirnov test and for homogeneity of variances with Levene test. Data were log transformed before analysis if data were not normally distributed. The *t*-test was performed to evaluate the differences between the training and validation group.

To conduct Shapley additive explanation (SHAP) analysis, several Python packages were utilized: SHAP, the primary library for calculating and visualizing SHAP values, offers interpretability for model predictions and highlights feature importance. Pandas, a robust library for data manipulation and preprocessing, facilitated the management of datasets, data cleaning, and preparation of inputs for SHAP analysis. NumPy, an essential package for numerical computations, provided support for array operations and the numerical calculations necessary for SHAP. Finally, Matplotlib, a versatile plotting library, was used to create various SHAP visualizations, including summary plots, bar plots, and waterfall plots, illustrating feature contributions to specific predictions.

## 3. Results

After exclusion for unfit subjects, a total of 907 participants (men: 481, women: 426) were enrolled with a complete 4-year follow-up data. The demographic data of the participants were shown in Table 2 (mean ± SD). We processed our variables by MLR first in Table 3 and found significant correlations about systolic blood pressure (*r* = −0.103, *P* = .004) and alanine aminotransferase (ALT) (*r* = 0.073, *P* = .029) with eGFR_end_.

**Table 2:** Demographic, biochemical, and lifestyle characteristics of the study participants.

Variables	Mean ± SD	n
Age (yr)	64.12 ± 11.91	860
BMI (kg/m^2^)	25.47 ± 3.66	224
Duration of diabetes (yr)	14.31 ± 8.25	860
Baseline urine microalbumin creatinine ratio	128.75 ± 495.37	799
Baseline glycated hemoglobin (%)	7.72 ± 1.58	897
Baseline triglyceride (mg/dL)	140.95 ± 106.13	903
Baseline high density lipoprotein cholesterol (mg/dL)	44.97 ± 12.53	654
Baseline low density lipoprotein cholesterol (mg/dL)	100.59 ± 28.53	893
Baseline alanine aminotransferase baseline (U/L)	29.11 ± 19.86	899
Baseline creatinine (mg/dL)	1.02 ± 0.47	907
Baseline systolic blood pressure (mm Hg)	130.44 ± 14.13	774
Baseline diastolic blood pressure (mm Hg)	75.04 ± 8.97	774
Estimated glomerular filtration rate after 4 years (mL/min/1.73 m^2^)	69.01 ± 28.61	907

	n (%)	N
Sex		907
Male	479 (52.8%)	
Female	428 (47.2%)	
Smoking		907
No	680 (75.0%)	
Yes	227 (25.0%)	
Alcohol		907
No	854 (94.2%)	
Yes	53 (5.8%)	

Continuous variables are presented as mean ± standard deviation; categorical variables are shown as number (percentage).

BMI = body mass index.

**Table 3:** Simple correlation coefficients between future estimated glomerular filtration rate and potential risk factors.

Age	Duration	BMI	SBP	DBP	HbA1c	TG	HDL	LDL	ALT	Cr
−0.25	−0.007	−0.075	−0.103*	−0.047	−0.001	−0.024	−0.014	0.044	0.073*	0.024

Correlation coefficients are shown for age, duration of diabetes, BMI, systolic blood pressure (SBP), diastolic blood pressure (DBP), glycated hemoglobin (HbA1c), triglycerides (TG), high-density lipoprotein cholesterol (HDL), low-density lipoprotein cholesterol (LDL), alanine aminotransferase (ALT), and creatinine (Cr).

BMI = body mass index.

*P < .05 was considered statistically significant.

As mentioned in the methods, 4 different errors were subsequently used to compare the accuracy between different methods (Mach-L and MLR) and the results were shown in Table 4. It should be pointed that the smaller these errors, the more precise of the methods. ANN had the larger errors than logestic regression and was excluded in the final analysis.

**Table 4:** Performance metrics of multiple linear regression and machine learning models for predicting estimated glomerular filtration rate.

	MAPE	SMAPE	RAE	RRSE	RMSE
MLR	0.338 ± 0.040	0.350 ± 0.036	1.130 ± 0.095	1.173 ± 0.075	33.656 ± 2.867
CART	0.270 ± 0.015	0.301 ± 0.020	0.999 ± 0.048	1.111 ± 0.052	31.903 ± 2.741
RF	0.268 ± 0.013	0.295 ± 0.015	0.985 ± 0.030	1.097 ± 0.025	31.536 ± 2.767
ANN	0.984 ± 0.002	1.935 ± 0.001	3.167 ± 0.178	2.585 ± 0.135	73.976 ± 2.169
XGBoost	0.298 ± 0.016	0.323 ± 0.021	1.053 ± 0.052	1.137 ± 0.046	32.662 ± 2.901

Values are presented as mean ± standard deviation. Models include multiple linear regression (MLR), classification and regression tree (CART), random forest (RF), artificial neural networks (ANN), and eXtreme Gradient Boosting (XGBoost). Metrics include mean absolute percentage error (MAPE), symmetric mean absolute percentage error (SMAPE), relative absolute error (RAE), root relative squared error (RRSE), and root mean squared error (RMSE).

The averaged importance percentage ranking of each potential risk factor generated by the RF, XGBoost, and CART methods was shown in Table 5. Different Mach-L models could provide distinct importance rankings of each risk factor.

**Table 5:** Ranking of risk factor importance derived from random forest, eXtreme Gradient Boosting, and classification and regression tree models.

	RF	XGBoost	CART	Average
Sex	65.41	50.35	2.89	39.55
Age	60.73	51.98	25.24	45.98
Duration of diabetes	80.33	47.05	19.44	48.94
BMI	66.47	67.77	100	78.08
SBP	48.08	42.52	35.46	42.01
DBP	59.23	30.63	34.58	41.48
Smoking	36.95	11.29	4.12	17.46
Alcoholic drinking	0.704	1.85	7.10	3.22
HbA1c	42.14	19.40	7.90	23.14
TG	60.48	55.6	1.23	39.10
HDL-C	69.11	49.85	35.68	51.54
LDL-C	89.78	55.07	2.48	49.11
ALT	65.99	28.89	4.87	33.25
Baseline Cr	19.73	3.44	0	7.72
MCR	83.34	49.85	19.21	50.80

The table shows the relative importance scores of each variable as estimated by different machine learning models. A higher average ranking is represented by a darker background color in risk factors.

ALT = alanine aminotransferase, BMI = body mass index, CART = classification and regression tree, Cr = creatinine, DBP = diastolic blood pressure, HbA1c = glycated hemoglobin, HDL = high-density lipoprotein cholesterol, LDL = low-density lipoprotein cholesterol, MCR = microalbumin creatinine ratio, RF = random forest, SBP = systolic blood pressure, TG = triglycerides, XGBoost = eXtreme Gradient Boosting.

**Figure 3.**
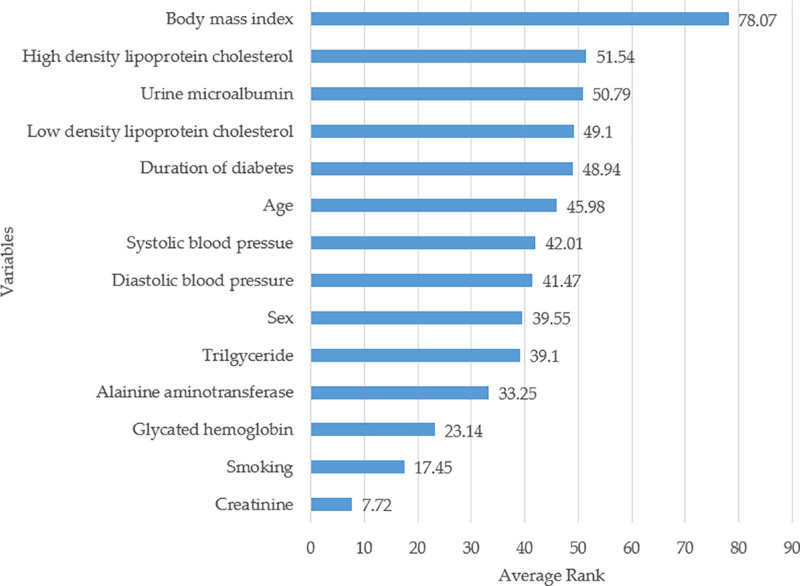
Ranking of risk factors based on machine learning model output. The figure displays the relative importance of risk factors as determined by the machine learning models.

We also averaged the rank value of each variable in each selected Mach-L method for the overall combining importance of each risk factor in predicting diabetes eGFR_end_. The increasing order was described in Figure 3. The first 6 important risk factors in predicting diabetic eGFR_end_ in a 4-year-follow-up cohort were BMI, baseline HDL-C, baseline microalbumin creatinine ratio (MCR), baseline LDL-C, duration of diabetes, and age.

**Figure 4.**
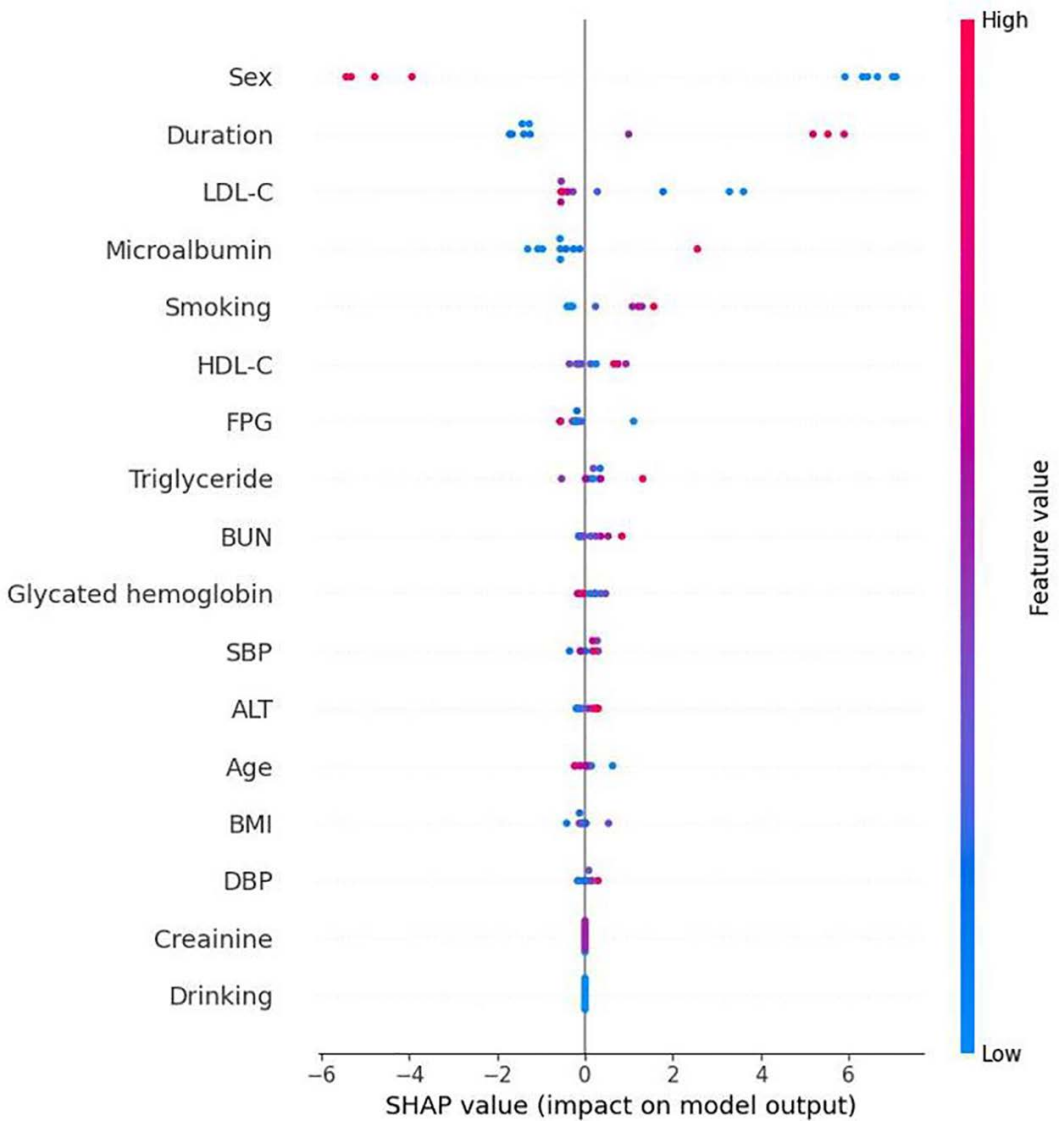
Summary beeswarm plot of Shapley additive explanation values. The figure presents the distribution and magnitude of SHAP values for features across the study cohort, indicating their contribution to the model output. ALT = alanine aminotransferase, BMI = body mass index, BUN = blood urea nitrogen, DBP = diastolic blood pressure, FPG = fasting plasma glucose, HDL-C = high-density lipoprotein cholesterol, LDL-C = low-density lipoprotein cholesterol, SBP = systolic blood pressure, SHAP = Shapley additive explanation.

**Figure 5.**
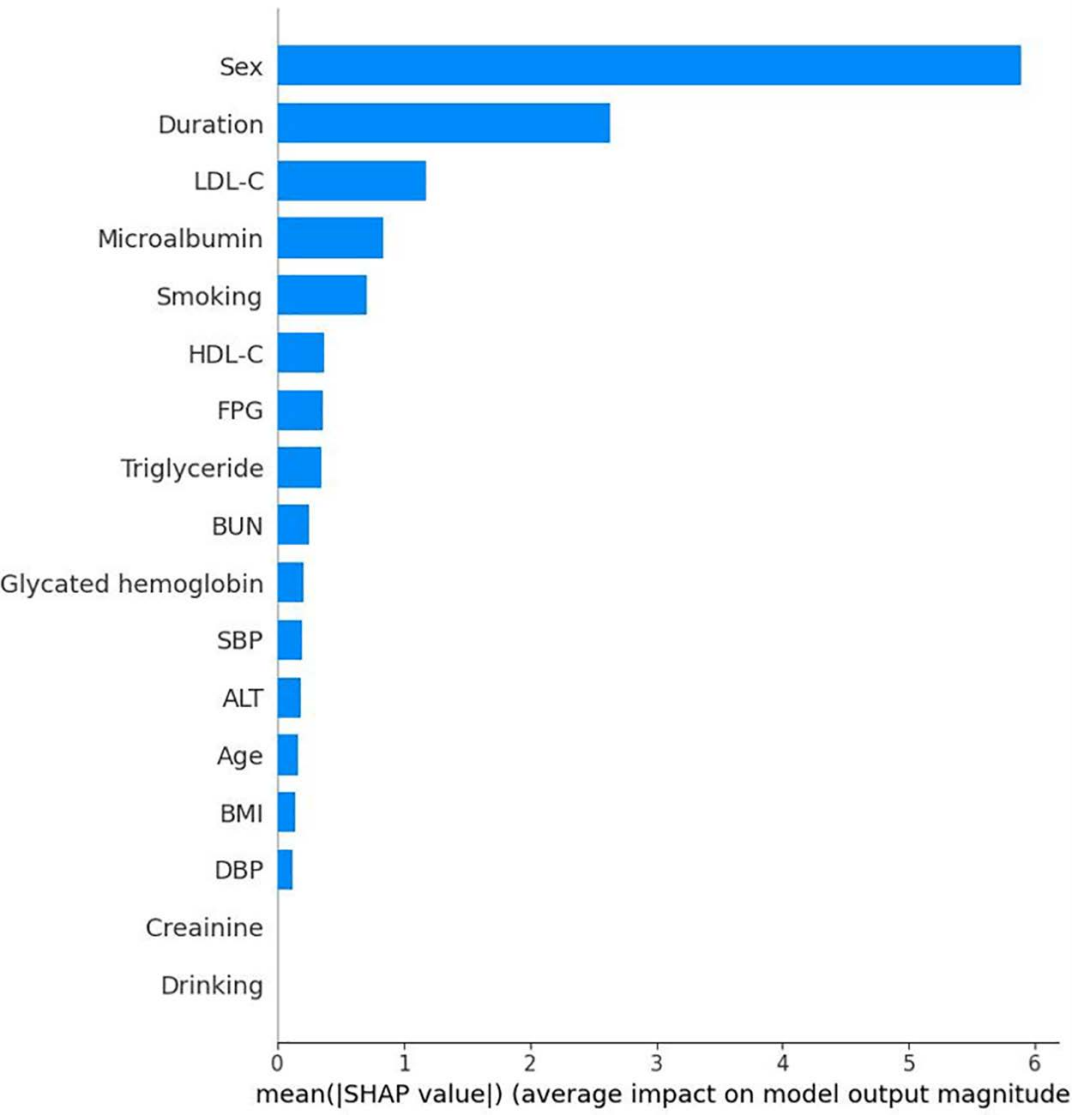
Bar plot of the absolute Shapley additive explanation values. The figure displays the mean absolute SHAP values of each feature, representing the overall impact on the model’s predictions. ALT = alanine aminotransferase, BMI = body mass index, BUN = blood urea nitrogen, DBP = diastolic blood pressure, FPG = fasting plasma glucose, HDL-C = high-density lipoprotein cholesterol, LDL-C = low-density lipoprotein cholesterol, SBP = systolic blood pressure, SHAP = Shapley additive explanation.

**Figure 6.**
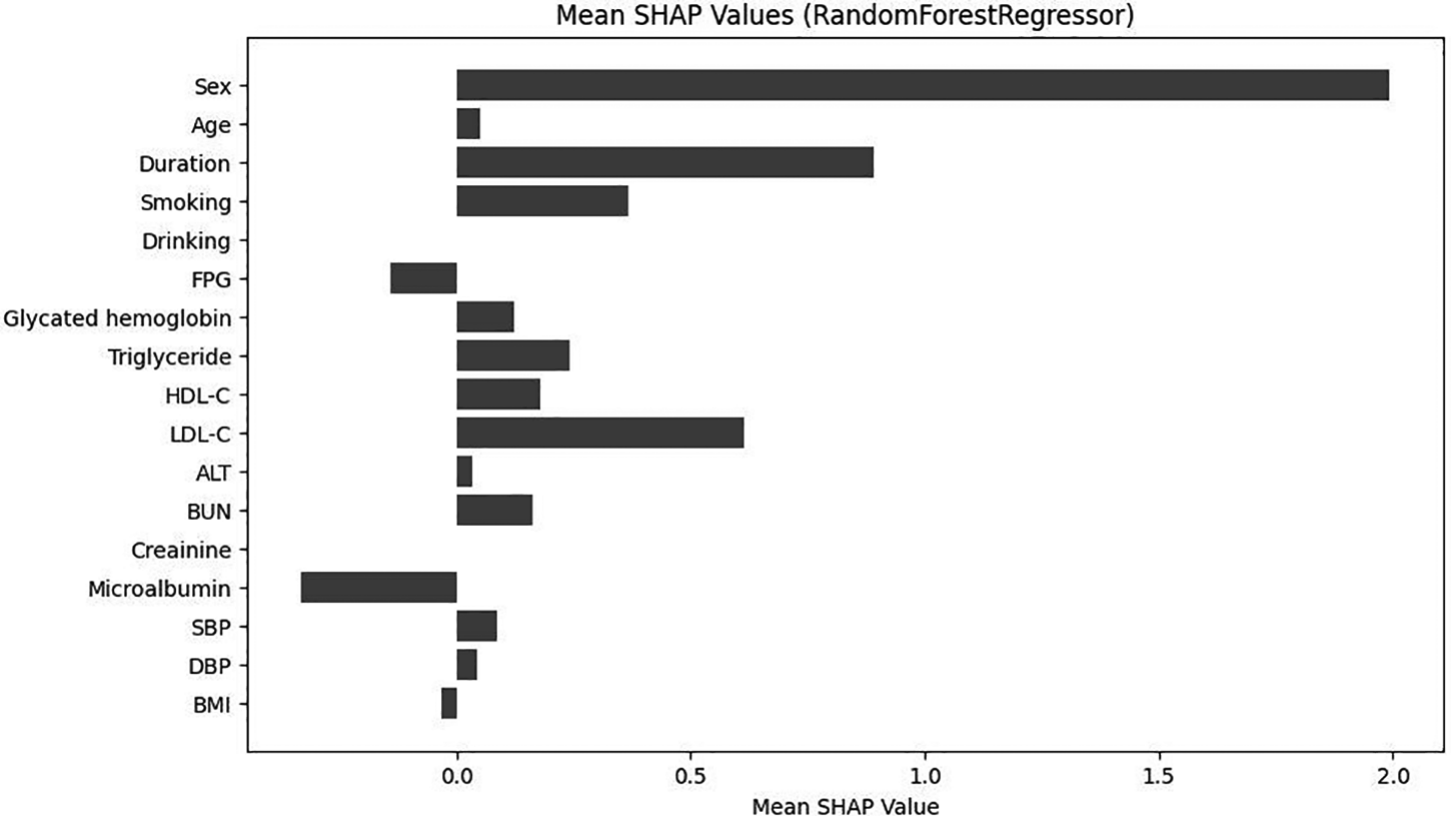
Plot of the true Shapley additive explanation values of the features. The figure shows the true SHAP values for each feature, illustrating their individual contribution to prediction outcomes. ALT = alanine aminotransferase, BMI = body mass index, BUN = blood urea nitrogen, DBP = diastolic blood pressure, FPG = fasting plasma glucose, HDL-C = high-density lipoprotein cholesterol, LDL-C = low-density lipoprotein cholesterol, SBP = systolic blood pressure, SHAP = Shapley additive explanation.

Each of the 4 Mach-L methods have their own SHAP. We presented the SHAP of RF to illustrate the value and direction of each feature since it has the smallest error calculated by the performance metrics. The SHAP results presented by summary plots were shown in Figure 4, and Figure 5 averages the absolute SHAP values from Figure 4, helping clinicians prioritize the features to be focused and to define the overall impact of a feature. Owing to the absolute values of the SHAP it did not show a positive or negative effect; Figure 6 showed the net values of SHAP (not the absolute values but the relative values) so the direction of the impacts for these features could be pointed. It could be noted that age, duration of diabetes, LDL, and HDL were positively correlated and BMI and MCR were negatively correlated to eGFR_end_.

## 4. Discussion

The present study has 2 main goals. The first was to compare the accuracies of 4 Mach-L methods with MLR in predicting eGFR_end_. The second was to determine and rank the importance of these risk factors and further determining the positive and negative impacts of each feature for eGFR_end_ in a Chinese T2D cohort followed up for 4 years. After modeling, our results showed that: RF, CART, and XGBoost were more superior than MLR in the prediction of the eGFR_end_; the most important 6 risk factors from the proposed model were baseline BMI, HDL-C, MCR, LDL-C, duration of diabetes, and age according to their importance of percentage; age, duration of diabetes, LDL, and HDL were positively correlated and BMI and MCR were negatively correlated to eGFR_end_.

The present study provided information about which factors had a more profound effects on the future eGFR which is novice and of clinical use.

In the following discussion, it should be noted that the abbreviation of CKD (chronic kidney disease) stands for renal disease in nondiabetic patients. In the same time, DKD represents otherwise deterioration of renal function in diabetic patients. Among all the risk factors, BMI was selected as the most important factor in present study. It is interesting to note that either T2D or obesity itself could lead to the deterioration of renal function.^[28,29]^ DKD is a well-known serious microvascular complication that is seen in about 40% of diabetes. In general, MCR occurs much earlier in the process of the eGFR deterioration and without any intervention in T2D; 20% to 40% with MCR progress to overt nephropathy after 20 years from the onset of diabetes and approximately 20% end-stage renal disease during their lifetime.^[29]^ On the other hand, the relationship of BMI and CKD was also well documented over the past 2 decades. Seeking for predictors of elevated creatinine level in people who were free of kidney disease at baseline, Framingham Offspring study cohort included participants from community-based sample without preexisting kidney disease, and after a mean follow-up period of 18.5 years, higher BMI was associated with higher risk of CKD [odds ratio = 1.23 per 1 SD; 95% confidence interval (CI): 1.08–1.41].^[28]^ The similar tendency could also be noted in obese T2D. In a study done in 1872 Taiwanese T2DM, Ou et al showed that obese people with BMI > 24 kg/m^2^ was significantly related to lower eGFR (<30 mL/min/1.73 m^2^) by using traditional MLR.^[30]^ Our result was in line with the other mainstay studies. The pathophysiology of obesity-related CKD is multifactorial. The direct impact of obesity is related to higher production of adiponectin, leptin, and resistin. Indirectly, the increased fat mass may cause hypertension and atherosclerosis.^[30]^ All of the above changes could deteriorate renal function via inflammation, abnormal lipid metabolism, oxidative stress, and renin-angiotensin-aldosterone system activation.^[31]^

It is well-known that in T2DM, higher triglyceride and lower HDL-C are the hallmarks of dyslipidemia.^[32]^ Interestingly, the lower HDL-C level was found to be correlated to lower eGFR. For example, in Action in Diabetes and Vascular Disease: Preterax and Diamicron Modified Release Controlled Evaluation (ADVANCE) study, 11,140 T2DM patients with at least 1 additional vascular risk factor were followed for 5 years. They confirmed lower baseline HDL-C level as a significant and independent predictor of the development of DKD at the end of follow-up.^[33]^ Another observational retrospective study was done by Russo et al which included 15,362 T2D and followed up for 4 years. The primary end point of DKD was defined as either low eGFR (<60 mL/min/1.73 m^2^) or an eGFR reduction >30% and/or albuminuria. At the end of the study, low HDL-C (<40 mg/dL in men, <50 mg/dL in women) was associated with a 27% increased risk of low GFR, and the risk of developing albuminuria was 24%, whereas the risk of developing both low GFR and albuminuria was 44%.^[34]^ Low HDL-C is one of the components of metabolic syndrome, which may lead to hyperglycemia and insulin resistance. The later contribute to subclinical inflammation and podocyte damage, and hyperinsulinemia may further cause glomerular hyperfiltration and increasing vascular permeability. These consequences of metabolic derangement precipitate the appearance of DKD and DKD progressing. The result of our study further strengthened the role of HDL-C in T2D as the second important factor for future eGFR.

The next risk factor is baseline MCR. This result is not surprising since the hallmark of established DKD is persistent albuminuria with coexisting retinopathy and no evidence of alternative kidney disease which is responsive for deteriorating eGFR.^[35]^ Most current observational studies have shown relationship between MCR and declining eGFR in T2DM patients and increasing albuminuria has generally been accepted as one of the first signs of DKD.^[36,37]^ Due to this, it remains the best documented predictor for high risk of development of diabetic nephropathy; the American Diabetes Association “Standards of Care in Diabetes” suggests urinary albumin and eGFR should be assessed at least annually in all patients with T2D regardless of treatment.^[38]^ Hyperglycemia increase mitochondrial superoxide production and dysregulates key intracellular metabolic pathways, thus leads to the production of material that directly cause glomerular endothelial cell dysfunction and also t. Recurrent and progressing of this scenario along with the development of other glomerular change further cause overt diabetic nephropathy.^[39]^

Modification of dyslipidemia has been recognized to reduce the rates of occurrence and progression of diabetic nephropathy.^[40]^ As part of diabetic dyslipidemia, it is not surprising that LDL-C was involved in the progression of DKD and being the 4th important factors for predicting eGFR_end_. Dejenie et al conducted a hospital-based comparative cross-sectional study of 104 patients with T2D and found LDL-C as being significantly associated with DKD (*P* < .05) in multivariable logistic regression analysis.^[41]^ Furthermore, statin therapy proved to be a potential effective choice for preventing DKD in T2D, according to a multicenter retrospective cohort study including 19,858 T2D patients without preexisting chronic kidney disease. In this cohort, statin initiation was associated with lower risks of DKD development (hazard ratio [HR] 0.72, 95% CI 0.62–0.83) and deteriorated kidney function (HR 0.60, 95% CI 0.44–0.81).^[42]^ Studies in T2D demonstrated that dyslipidemia stimulate inflammatory pathways via the secretion of proinflammatory cytokines, causing the production of reactive oxygen species, which lead to excessive extracellular matrix production in the glomeruli, and resulting in damage to the glomeruli and glomerular glycocalyx.^[43]^

Duration of diabetes is an important factor to deteriorating renal function. For instance, Kaewput et al analyzed a nationwide, multicenter, observational cohort of 8464 T2D. They followed the patients for 29 months and analyzed risk factor using Cox proportional hazard model. Increased duration from diagnosis of T2DM is considered as a risk factor associated with progression to CKD stage 5 or the need for chronic dialysis (HR: 1.17 per 5-year increase, 95% CI 1.01–1.35, *P* = .04).^[44]^ In the same time, by using The Action to Control Cardiovascular Risk in Type 2 Diabetes (ACCORD) clinical trial dataset, Buyadaa et al found that younger age-of-onset or longer diabetes duration had more rapid deterioration of eGFR, comparing with patients diagnosed in middle age or with shorter duration of diabetes among T2DM patients.^[45]^ Our results are in line with other cornerstone studies that the duration of diabetes was the 4th important factor to influence eGFR_end_. Our result showed that the longer the duration of diabetes, the more severe nephropathy became. The possible pathophysiology of greater annual eGFR decline along with increasing duration of diabetes maybe the hyperfiltration and its subsequent normalization in people with younger onset of diabetes. Also, it cannot be excluded that elements related to the feature of obesity in these group, such as elevated fatty acids and leptin, may contribute to greater decline in kidney function by increased oxidative stress. Genetic predisposition, faster beta cell function decline, increased insulin resistance, more vulnerable kidney tissue, and other unknown factors may also play an important role of more rapid development of renal insufficiency.^[45,46]^

Finally, aging is well known to decrease eGFR in healthy population.^[47]^ However, owing to renal function dose not deteriorated obviously until the mid-70s, other factors might accelerate the natural course of declining of the eGFR in adults.^[47]^ In T2D, similar findings were reported by Zoppini et al and Rossing et al that age was an independent predictor of annual eGFR decline by MLR analysis in their longitudinal studies.^[48,49]^ Recently, Svensson et al also conducted a prospective cohort study with 794 Swedish people, which confirmed that older age when diabetes is diagnosed was a significant and independent predictor (*P* = .030) of DKD development over 17 years diabetes duration.^[50]^ Similar finding was also reported by a National registry study from 1,113,201 people in Australia, which recorded the incidence of DKD was higher in those with an older age of onset of diabetes in the first 20 years following the onset of diabetes.^[51]^ All these findings supported our result that age was negatively related to eGFR_end_. The effects of aging on decreasing eGFR was no doubt multifactorial. It could be partially attributed to previously mentioned risk factor of deteriorated renal function with simply increasing age.^[47]^ Those who were diagnosed with T2D later in life were also more likely to appear with nephropathy when diabetes was noted, including caused by condition other than T2D. Furthermore, it was reasonable to surmise that hypertension, a well-known risk factor of DKD, existed more universally in the older age people and could accelerate DKD progressing.^[51]^

According to our data analysis by using MLR, ALT was significantly associated with future eGFR (*r* = 0.073, *P* = .029). This is supported by Adiga and Malawadi, who found that higher ALT level reported to be associated with better renal functions (*r* = 0.439, *P* = .0001).^[52]^ Also, in a retrospective study of 690 Japanese T2D patients, it appeared that the likelihood of DKD regression was higher in patients with higher serum ALT level (95% CI 1.002–1.018; *P* < .005; 1 IU/L).^[53]^ However, the pathophysiological relationship of serum ALT and DKD is still controversial. Uremic toxins itself may limit the activity of the ALT enzyme. Other possible mechanisms include reduction in pyridoxal-5-phosphate, a coenzyme of aminotransferase, the presence of ultraviolet absorbing material, and decreased synthesis and inhibition of releasing AST and ALT from hepatocytes.^[54]^ In our analysis as well, when ALT was high, eGFR was less likely to worsen in the future. The significant positive correlation between ALT and eGFR highlights the importance of liver disease such as nonalcoholic fatty liver disease in T2D.

This study had some limitations. First, we did not collect information on the use of angiotensin-converting enzyme inhibitors, angiotensin receptor blockers, sodium-glucose cotransporter 2 inhibitors, and glucagon-like peptide-1 agonists. All these medications would have beneficial effects on eGFR decline. Second, the smoking and alcohol details need to be more specific. Even though these drawbacks do exist, our large number and the characteristics of Mach-L could adjust these drawbacks. Thirdly, the present study was done only in Chinese, therefore, extrapolation to other ethnic group should be exercised with caution. Fourth, the patients were recruited from who visited hospital for T2D control and therefore may underrepresent those who manage diabetes with exercise and diet alone. And last, according to the results of simple correlation (Table 3) and the net values of SHAP (Fig. 6), it could be noted that the positive and negative influence of HDL, duration of diabetes, and age were not in the same way. It is not easy to give an account at present for the differences. Future studies should validate these findings in diverse racial/ethnic populations and explore the integration of Mach-L algorithms into routine clinical practice for DKD risk prediction.

## 5. Conclusions

Mach-L methods were proved to be more accurate in predicting eGFR_end_ than traditional MLR. By using 3 different Mach-L methods, our results showed that BMI was the most influential factor for eGFR_end_, followed by baseline HDL-C, baseline urine MCR, baseline LDL-C, duration of diabetes, and age in a Chinese T2DM cohort followed up for 4 years. In these risk factors, age, duration of diabetes, LDL, and HDL were positively correlated and BMI and MCR were negatively correlated to eGFR_end_. Our findings underscore the clinical utility of Mach-L in predicting renal function decline, which may inform proactive interventions in T2D management.

## Author contributions

**Conceptualization:** Fang-Yu Chen, Dee Pei, Chun-Heng Kuo, Li-Ying Huang, Mao-Jhen Jhou, Yao-Jen Liang.

**Methodology:** Mao-Jhen Jhou.

**Supervision:** Yao-Jen Liang.

**Validation:** Li-Ying Huang.

**Writing – original draft:** Fang-Yu Chen.

**Writing – review & editing:** Dee Pei, Yao-Jen Liang.
